# An Optimization-Based Orchestrator for Resource Access and Operation Management in Sliced 5G Core Networks

**DOI:** 10.3390/s22010100

**Published:** 2021-12-24

**Authors:** Chiu-Han Hsiao, Yean-Fu Wen, Frank Yeong-Sung Lin, Yu-Fang Chen, Yennun Huang, Yang-Che Su, Ya-Syuan Wu

**Affiliations:** 1 Research Center for Information Technology Innovation, Academia Sinica, Taipei 11529, Taiwan; yennunhuang@citi.sinica.edu.tw; 2 Graduate Institute of Information Management, National Taipei University, New Taipei City 237303, Taiwan; yeanfu@mail.ntpu.edu.tw; 3 Department of Information Management, National Taiwan University, Taipei 10617, Taiwan; flin@ntu.edu.tw (F.Y.-S.L.); D09725003@ntu.edu.tw (Y.-F.C.); R06725052@ntu.edu.tw (Y.-C.S.); R06725016@ntu.edu.tw (Y.-S.W.)

**Keywords:** sliced 5G core network, orchestrator, access control, resource scheduling, migrations, lagrangian relaxation

## Abstract

Network slicing is a promising technology that network operators can deploy the services by slices with heterogeneous quality of service (QoS) requirements. However, an orchestrator for network operation with efficient slice resource provisioning algorithms is essential. This work stands on Internet service provider (ISP) to design an orchestrator analyzing the critical influencing factors, namely access control, scheduling, and resource migration, to systematically evolve a sustainable network. The scalability and flexibility of resources are jointly considered. The resource management problem is formulated as a mixed-integer programming (MIP) problem. A solution approach based on Lagrangian relaxation (LR) is proposed for the orchestrator to make decisions to satisfy the high QoS applications. It can investigate the resources required for access control within a cost-efficient resource pool and consider allocating or migrating resources efficiently in each network slice. For high system utilization, the proposed mechanisms are modeled in a pay-as-you-go manner. Furthermore, the experiment results show that the proposed strategies perform the near-optimal system revenue to meet the QoS requirement by making decisions.

## 1. Introduction

Network slicing is a novel technology starting from the fifth-generation (5G) mobile communication networks which has high capacity, high data rate, high energy efficiency, and low end-to-end (E2E) delay [1]. The system architecture adopts the virtualization techniques to make the virtualized network functions (VNF) dynamically allocated to servers in a cloud computing environment [2,3,4,5,6]. Software-defined networking (SDN) is an enabling technology for the network access management, deployment, configuration, and control of the data forwarding planes of the underlying resources [7,8,9,10]. Each slice has the logical and independent network corresponding to the quality of service (QoS) requirements for applications, such as the Internet of Things (IoT) for massive machine-type communication (mMTC) [11,12], emerging AR/VR media applications, UltraHD, or 360-degree streaming video for enhanced mobile broadband (eMBB) [6], and industrial automation, intelligent transportation, or remote diagnosis and surgery for ultra-reliable low-latency communication (URLLC) [13]. Furthermore, the challenging research problems are derived and discovered in the following aspects:

1. A well-designed system architecture for 5G slices that can efficiently handle diversified services and maximize system resource utilization is required.
2. Network management’s scalability and efficiency should be considered carefully with the rapid changes of user demands, such as mobility, time-varying conditions, traffic load distributions, etc. [13].
3. A sophisticated resource orchestrator for network operation should be designed for the network deployment and management of resources by access control, scheduling, and migrations for serving multi-variant types of services, achieving application service differentiation, or maintaining massive connectivities [13,14].

**Figure 1:**
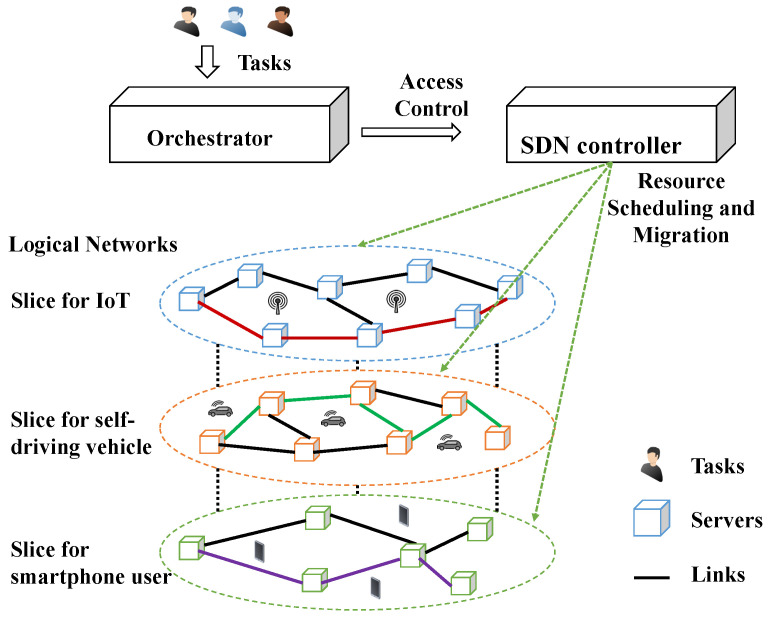
System architecture for orchestrator in a sliced network.

Operators always desire to develop a sustainable network with appropriate network access management mechanisms [9]. A sophisticated resource orchestrator for resource allocations is a promising approach that can improve QoS and energy efficiency shown in Figure 1. For pursuing a positive user experience, many operators also switch their focus on how to improve the QoS [15]. An orchestrator for network operation implemented by applying resource scheduling and resource allocation strategies is crucial [6,13,16].

The critical factors of resource management, such as access control, scheduling, and migrations, are analyzed in this paper on flexibility and scalability over cloud computing in sliced network. However, the network resource allocation based on data traffic and network performance is deployed by slices with heterogeneous QoS satisfied 5G traffic management characteristics. The optimal resource allocation algorithms embedded in an orchestrator are also implemented to optimize resource utilization in the end-to-end slices as the objective function to maximize the system value [5,17,18,19].

To address the challenges mentioned above, this study’s purpose and scope are proposed to stand on an ISP to optimize system resource utilization in operational stages. The system architecture is shown in Figure 1. The orchestrator with a Lagrangian-Relaxation-based solution approach is a coordinator with network resource operation and scheduling to deal with resource management problems. It offers reliable QoS to users and provides flexibility and scalability over a cloud-based environment. A centralized computing resource management model is formulated as a mathematical programming problem to optimize the total system revenue through access control, weighted task scheduling, server operation, and resource migration. This work analyzes how the core networks acquire resources to satisfy various user requirements subject to budget and QoS constraints. The objectives are to find efficient and effective ways of obtaining the primal feasible solutions to the optimal.

Furthermore, the operation is divided into slices with many time slots concerned in the evaluation period—additionally, the available hosts scheduling with migration to adjust into active hosts. The operation costs are reflected in the objective function. Based on enough system capacities, the task processes with the QoS constraints are considered to satisfy user experience. However, the extra migration effort and active hosts are calculated as a trade-off problem to migrate resources or shutdown hosts for power saving in limited resources. Accordingly, the problem formulation is extended from previous work [20]. The objective function includes access control variables for task assignment, host on-off variables, and migration variables to maximize system revenue with the proposed scheduling heuristics among allocated time slots. Several constraints are formulated according to the research problem. The computational experiments and the scenarios are designed for an orchestrator for network operation to show how the experimental cases lead to a real-life situation. Then, the proposed heuristics can then iteratively obtain the primal feasible solutions with LR and dual problems to effectively minimize the solutions’ quality (GAP). The algorithms are embedded and implemented into the orchestrator to significantly, efficiently, and effectively apply resource scheduling and resource allocation strategies. This work is to concentrate on the detailed operation for practical evaluation. The main contributions of this work compared briefly to the previous work [20] include.

The reminder of this paper is organized as follows. The literature review of the existing ideas and mechanisms for an orchestrator designed in the 5G network is presented in Section 2 from communication and computation perspectives. In Section 3, the problem definitions for the resource management are described, and a mathematical programming model is formulated. Section 4 presents the proposed solution through bin-packing algorithms as initial solutions, Lagrangian Relaxation (LR) methods and optimization-based heuristics (SP, UP, and OI) developed to determine the primal feasible solutions. Section 5 presents various computational experiments, and the results are correspondingly discussed and validated to support the proposed mechanisms. Finally, the discussion and conclusions based on numerical results are drawn for the orchestrator design proposed for network operation, and the future work is described in Section 6.

## 2. Related Work

### 2.1. Communication Perspective

The coordinated allocation of communication resources to improve the QoS is a critical task [21]. Zhai et al. proposed resource allocation algorithms to manage the utilization of servers under burst and variant traffic conditions [22]. To improve the resource pool utilization and spectrum efficiency, Lu et al. proposed a dynamic resource allocation mechanism based on a Karnaugh map and genetic algorithm [23]. A multi-resource allocation model based on a semi-Markov decision process (SMDP) was developed by Liu et al. [21]. The problem was solved by linear programming techniques to attain resource allocation decisions in cloud computing. In addition, by assuming that all clouds can provide the same resources and get different rewards, Jin et al. [24] designed an auction mechanism to allocate resources to match the capacity of users’ demands in a cloud computing environment appropriately. In this auction model, mobile devices are simulated to the buyers and cloudlets are simulated to the sellers. The auctioneer is the centralized control manager. Its responsibility is to drop the transmission costs and latency between mobile devices and clouds. Table 1 shows the studies on resource allocation in a cloud computing environment.

Related works are linked to some findings addressed in this paper. From the communication perspective, the methods or algorithms of an orchestrator focus on delivering satisfactory QoS in the communication perspectives. An orchestrator’s system scalability and flexibility in resource allocation are also addressed. The operation costs are reflected in the objective function. The task processes with the QoS constraints are considered to satisfy user experience. The extra migration effort and active hosts are calculated as a trade-off problem to migrate resources or shutdown hosts for power saving. Accordingly, the problem formulation is extended from previous work [20]. The objective function includes access control variables for task assignment, host on-off variables, and migration variables to maximize system revenue with the proposed scheduling heuristics among allocated time slots. Accordingly, the proposed model and constraints address the relevant issues of scalability and flexibility in resource allocation. The objective is to use the proposed heuristics to maximize the total value of the task to properly schedule or rearrange resources to fulfill user requirements.

### 2.2. Computational Perspective

Online VM placement algorithms for allocating resources to VMs in a cost-effective manner were proposed in Reference [25] for increasing revenue. First-fit (FF), first-fit-migration (FFM), least reliable first (LRF), and decreased density greedy (DDG) algorithms were presented for examining the packing strategies. The bin-packing problem was proved to be an NP-complete problem [26]. Those methods can pack most of the tasks to be the performance metrics [25]. In the case of 5G C-RAN, the VM migration between servers is allowed [2,3,4]. However, VM scheduling, including migrations, is a crucial method to increase system utilization [27]. VM migration and scheduling overheads should be considered for overall sustainable network evolution, such as access control, resource allocation, scheduling, and migrations in a cloud-based environment. The extra migration effort and active hosts are calculated as a trade-off problem to migrate resources or shutdown hosts for power saving. Accordingly, the problem formulation is extended from previous work [20]. The unbalanced supply and demand inspire this study during peak traffic hours. The objective is to select valuable tasks to achieve maximum revenue subject to task assignment and limited migrations in a limited resource pool. The problems are well-known classified as bin-packing and 0/1 knapsack problems. Herein, access control and resource scheduling are jointly considered for network operation. The decisions are designed in three dimensions, tasks, time, and servers. For example, the task should be assigned or not and to which server at which time slot. In order to fit the QoS requirements, the orchestrator is embedded with LR-based assignment and scheduling strategies, such as computing and transmission in variant traffic loads in a global view adopted by heuristics bin-packing strategies.

### 2.3. Motivation and Research Scope

The related work summarizes that 5G is expected to deliver high QoS to users for emerging applications. For pursuing a positive user experience and maximize system revenue, a set of resource optimization management questions is proposed for the design of an orchestrator on network operation, and the approach is also examined by considering various constraints and goals for the improvement of QoS and system revenue in 5G C-RAN shown in Table 1. The problem is combined with Knapsack problem and bin-packing problems, which are NP-complete problem, and it is not easy to solve by standard tools or methods in polynomial time. To deal with the problem, a Lagrangian-Relaxation-based solution approach with high efficiency and effectiveness is proposed. Before introducing the solution approach, a precise mathematical programming model of centralized management is required relevantly. The formulation should have a well-modeled mathematical structure and formulate the behaviors from an operator perspective to offer higher QoS for users in the networks. A sophisticated modeling technique should be applied correctly for the model. Then, the solving procedures with the Lagrangian-Relaxation-based method could be applied. Based on the mathematical structure, the primal problem could be decomposed by relaxing constraints. It benefits the solution approach to obtain primal feasible solutions reasonably compared with standard or general-purpose methods. Each sub-problem could be easily solved by algorithms or heuristics in a few steps optimally. The lower bounds can be determined and rigorously used to evaluate solution quality (GAP) proposed in this paper. One of the contributions is that applying multipliers’ characteristics in the LR problem presented as significant indices presenting to find the optimal solution systematically. The performance of the proposed indices with heuristics (SP, UP, and OI) and classical methods (FF and CP) is evaluated in different cases for determining operating decisions within some experimental conditions. The proposed algorithms provide a significant guideline to efficiently and effectively obtain optimal or near-optimal solutions with the well-defined mathematical programming model.

## 3. System Model and Mathematical Formulation

Figure 1 illustrates an abstract system model in an optimization-based framework. The tasks are represented as user computing demands concerning values, amount of computational resources, processing time, and delay tolerance. The heterogeneous servers have various levels of limited computational resources. For example, an application requests diverse computational resources simulated as tasks for transmission and data processing. The orchestrator has to make acceptance or rejection to the user requirements. During the time slot after arrival and before departure, the computational resources are all reserved and allocated to resource pools even with operations in a sliced network, such as VM migrations or turning on new servers. Furthermore, traffic loads vary over time. The research problem is how to manage resources of tasks for VM allocations and system utilizations. The task access control, computing resource allocation, switching servers on and off, and task migrations are implemented as the objectives of the orchestrator design.

The research problem is formulated as a mathematical programming problem. It attempts to involve a higher amount of VMs with VNFs emulated as tasks to maximize system revenue. However, the supplied resources and required demands are not equivalent. It forces a trade-off while deciding the tasks selected with maximum values and assigned them to servers with limited capacities through the proposed arrangement algorithms. For the finite number of tasks and servers, each VM is simulated as a task requested a CPU processing power and memory space package. Each server has the finite CPU processing power and memory for packing VMs. Each server incurs a start power cost when it is switched on. The delay in VM migration between servers is a type of cost. At least one server must be maintained active to accept VMs during the operating periods. The system revenue is set as an objective function. Algorithms of the orchestrator are designed to rely on decision variables, such as access control, resource allocation, server operation (switching on and switching off), and VM migrations. The access control and resource allocation decisions are jointly considered in three dimensions including tasks, time, and servers. The objective function is to maximize the total profits by admitting tasks subject to the budget of operating servers and migrating VMs in C-RAN. The constraints are jointly considered with task assignment, server capacity, server switching on or off, and VM migration. The given parameters and the decision variables used in this work are listed in Table 2 and Table 3, respectively.

The objective function is formulated as an integer programming (IP) problem to optimize the system revenue of the entire system for network operation. The objective function, $Z_{\mathrm{IP}}$, comprises the values corresponding to the maximum tasks assigned to subtract the setup cost of servers in C-RAN.

The first term of the objective function represents the total values of served tasks in the system. $V_{i}$ is the reward rate of task *i*. A task blocking variable, $b_{i}$, is set for the task rejection into the system in the second term of the objective function. The penalty, $N_{i}$, is considered if a task is not admitted to be processed due to the task requests are not entirely served by task assignments. The penalty values are set to be higher than the values of the reward rate. There are three types of cost: initial cost rate $A_{s}$, reopening cost $E_{s}$, and migration cost $F_{i}$. $A_{s}$ is the estimated cost per unit time for server *s*. The total initial cost is estimated based on the on-going time slots, which is referred to Amazon EC2 operation. $E_{s}$ is charged for a server to be switched on one at a time after server initialization. The value of $E_{s}$ is designed to be greater than that of $A_{s}$ in the experiments. The migration cost occurs when a VM process migrates to other servers. It represents a type of overhead of computational resource disturbances. The difference between time index τ and π is the time scale, and τ is a superset of π. The approach tries not only to get the maximum values of assigned tasks efficiently but also to control the servers in a cost-effective way in the network operation stage. The mathematical programming problem is shown as follows:

(1) $$\begin{array}{cc} Z_{\mathrm{IP}}=\; & \sum_{\tau \in T}\sum_{i\in I}\sum_{s\in S}V_{i}a_{\tau is}-\sum_{\tau \in T}\sum_{s\in S}A_{s}x_{\tau s}\\ & -\sum_{\tau \in T}\sum_{s\in S}E_{s}y_{\tau s}-\sum_{\pi \in \Pi }\sum_{i\in I}F_{i}\eta_{\pi i}-\sum_{i\in I}N_{i}b_{i} \end{array}$$

$$\begin{array}{cc} \; & \mathrm{Objective}\;\mathrm{function}\text{:}\\ \; & \max Z_{\mathrm{IP}}=\min-Z_{\mathrm{IP}}\\ \; & \mathrm{subject}\;\mathrm{to}\text{:}\\ \; & \;\left(C.\;1\right):\;\sum_{s\in S}a_{\tau is}\leq 1,\;\;\;\;\;\;\;\;\;\;\;\;\;\forall \tau \in T,\;\forall i\in I,\\ \; & \;\left(C.\;2\right):\;\sum_{s\in S}\sum_{\tau \in T}a_{\tau is}\leq \gamma_{i},\;\;\;\;\;\;\;\;\;\;\forall i\in I,\\ \; & \;\left(C.\;3\right):\;a_{\tau is}\leq x_{\tau s},\;\;\;\;\;\;\;\;\;\;\;\;\;\;\forall \tau \in T,\;\forall i\in I,\;\forall s\in S,\\ \; & \;\left(C.\;4\right):\;\delta_{i}\leq \sum_{s\in S}a_{\tau is}t_{\tau },\;\;\;\;\;\;\;\;\;\;\;\;\forall \tau \in T,\;\forall i\in I,\\ \; & \;\left(C.\;5\right):\;\sum_{s\in S}a_{\tau is}t_{\tau }\leq \delta_{i}+\gamma_{i}+\epsilon ,\;\;\;\;\;\;\forall \tau \in T,\;\forall i\in I,\\ \; & \;\left(C.\;6\right):\;\frac{\gamma_{i}-\sum_{\tau \in T}\sum_{s\in S}a_{\tau is}}{\gamma_{i}}\leq b_{i},\;\;\;\;\;\;\;\forall i\in I,\\ \; & \;\left(C.\;7\right):\;b_{i}\leq \gamma_{i}-\sum_{\tau \in T}\sum_{s\in S}a_{\tau is},\;\;\;\;\;\;\;\forall i\in I,\\ \; & \;\left(C.\;8\right):\;\frac{\sum_{i\in I}b_{i}}{\left|I\right|}\leq K,\\ \; & \;\left(C.\;9\right):\;\sum_{i\in I}a_{\tau is}D_{i}\leq x_{\tau s}P_{s}C_{s},\;\;\;\;\;\;\;\forall \tau \in T,\forall s\in S,\\ \; & \;\left(C.\;10\right):\;\sum_{i\in I}a_{\tau is}R_{i}\leq x_{\tau s}M_{s},\;\;\;\;\;\;\;\forall \tau \in T,\;\forall s\in S,\\ \; & \;\left(C.\;11\right):\;G\leq \sum_{s\in S}x_{\tau s},\;\;\;\;\;\;\;\;\;\;\;\;\;\forall \tau \in T,\\ \; & \;\left(C.\;12\right):\;x_{\tau s}-x_{(\tau -1)s}\leq y_{\tau s},\;\;\;\;\;\;\;\;\forall \tau \in T,\;\forall s\in S,\\ \; & \;\left(C.\;13\right):\;0\leq \sum_{\tau \in T}y_{\tau s},\;\;\;\;\;\;\;\;\;\;\;\;\;\forall s\in S,\\ \; & \;\left(C.\;14\right):\;\sum_{\tau \in T}y_{\tau s}\leq \frac{T}{2},\;\;\;\;\;\;\;\;\;\;\;\;\forall s\in S, \end{array}$$

$$\begin{array}{cc} \; & \;\left(C.\;15\right):\;a_{\tau is}\leq \alpha_{\pi is},\mathrm{for}t_{\pi -1}<t_{\tau }<t_{\pi },\;\forall \tau \in T,\;\forall i\in I,\;\forall s\in S,\;\forall \pi \in \Pi ,\\ \; & \;\left(C.\;16\right):\sum_{s\in S}\alpha_{\pi is}\leq 1,\;\;\;\;\;\;\;\;\;\;\;\;\;\forall \pi \in \Pi ,\;\forall i\in I,\\ \; & \;\left(C.\;17\right):\alpha_{\pi is}-\alpha_{(\pi -1)is}\leq \eta_{\pi i},\;\;\;\;\;\;\;\forall \pi \in \Pi ,\;\forall i\in I,\;\forall s\in S. \end{array}$$

where (C. 1)–(C. 8) are task assignment constraints, (C. 9)–(C. 10) are capacity constraints, and (C. 11)–(C. 17) are switching constraints.

For task assignment constraints, the admitted task *i* should be assigned to one of the servers. The summation of $a_{\tau is}$ overall servers should not be greater than one, as shown in constraint (C. 1). Constraint (C. 2) represents that the processing time slot requested by task *i* should be satisfied. The total assignment time slots are equal to the requested time slot. Constraint (C. 3) implies that only if task *i* is assigned to server *s*, server *s* must be switched on. Accordingly, both decision variables $a_{\tau is}$ and $x_{\tau s}$ are set to one. If task *i* is not assigned to any server, $a_{\tau is}$ is set to zero, and $x_{\tau s}$ can be a free variable. Constraint (C. 4) and (C. 5) imply that time is assigned for task *i* in chronological order. More precisely, task *i* should be processed after it arrives and needs to be earlier than its deadline. The deadline is presented by the delay tolerance, which begins in arrival time. Constraints (C. 6) and (C. 7) represent the correlation between task required time slot for assignments and the task blocking decisions. The blocking decision $b_{i}$ is set to one when the requested time slots of $\gamma_{i}$ of processing are not satisfied. Otherwise, the decision is set to zero. Constraint (C. 8) presents that the total rate of blocking tasks should not exceed that of the system requirement. *K* represents the system requirement of total task-blocking rate, for example, 3%, which implies that fewer than 3% tasks will be blocked.

For capacity constraints, constraint (C. 9) and (C. 10) are intuitively obtained by the total requirements of tasks aggregated in server *s*. The aggregated traffic should not exceed the server capacity. To put it another way, if a new amount of traffic load (CPU or memory) is arriving and is assigned to server *s*. After that, it should not be greater than either the remaining CPU or memory capacities of server *s* [28].

Constraint (C. 11) is associated with a policy of system design about the number of reserved servers. That is, the number of power-on servers should be at least greater than the minimum amount, *G*, for each time slot of τ. Constraint (C. 12) shows that the decision variable $y_{\tau s}=1$ is determined by $x_{\tau s}-x_{(\tau -1)s}$, which implies server *s* is powered on in the time slot τ.

**Figure 2:**
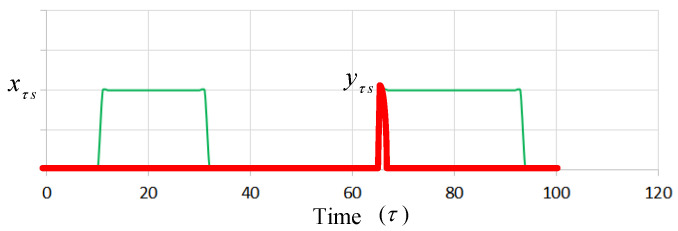
Presentation of server switching on or off.

Figure 2 presents an example in which the green dotted curve is represented as $x_{\tau s}$. The red pulse line, which is represented as $y_{\tau s}$, is re-switched on within the time slot (τ = 66). Constraints (C. 13) and (C. 14) imply that the total number of time slots the server switches on or off should not exceed the boundary condition during a time slot period. A faulty server may be caused if the operating time is over the boundary, which is $\frac{T}{2}$ in an assumption of the proposed model. Constraint (C. 15) implies that if task *i* is assigned to server *s* in time slot τ, then the parameter $\alpha_{\pi is}$ is set to one. The time index τ is a superset of π; that is, each time slot π contains one or more time slots τ, if a $a_{\tau is}$ in this time slot is set to one, $\alpha_{\pi is}$ is also set to one.

**Figure 3:**
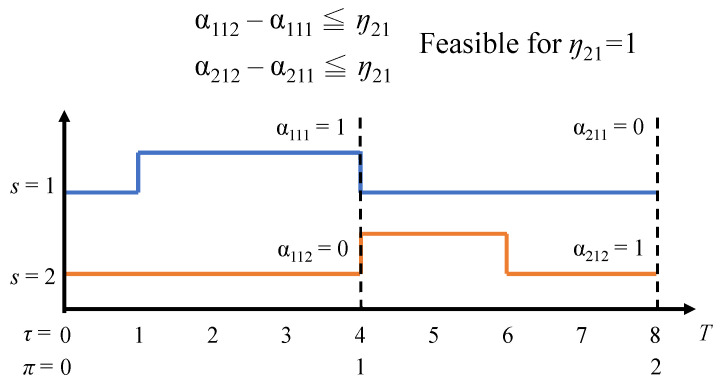
Presentation for task assignment and migration.

For Constraint (C. 16), each task should be only served in one server in the time slot π. Constraint (C. 17) implies the decision variable $\eta_{\pi i}$ is determined by $\alpha_{\pi is}-\alpha_{(\pi -1)is}$. Figure 3 shows an example for calculating $\eta_{21}$ for task 1 with two servers, and we can observe that the $\eta_{21}$ is set to one due to the migration occurs.

## 4. Lagrangian Relaxation-Based Solution Procedures

The LR approach is proposed to solve large-scale mathematical programming problems in the 1970s [29]. This approach has become a procedure for dealing with mathematical programming problems, such as integer or nonlinear programming problems, in many practical situations.

At the beginning, the idea is to relax complicated constraints into the primal problem and extend feasible solution regions to simplify the primal problem. The primal problem is transformed into an LR problem which is associated with Lagrangian multipliers [20]. Then, the LR problem can be divided into several independent sub-problems using decomposition methods associated with their decision variables and constraints. By applying the LR method and the subgradient method to solve the problems, some heuristics are designed rigorously to solve each sub-problem optimally. We can determine theoretical lower bounds from the primal feasible solutions and find helpful hints for obtaining the primal feasible solutions.

Suppose that the set of suboptimal solutions is satisfied using the original constraints. In that case, the solutions are known as primal feasible solutions. A near-optimal primal feasible solution has to be found to solve the primal problem. Thus, the objective values of the primal problem are iteratively updated after sequentially feasible verifications.

**Figure 4:**
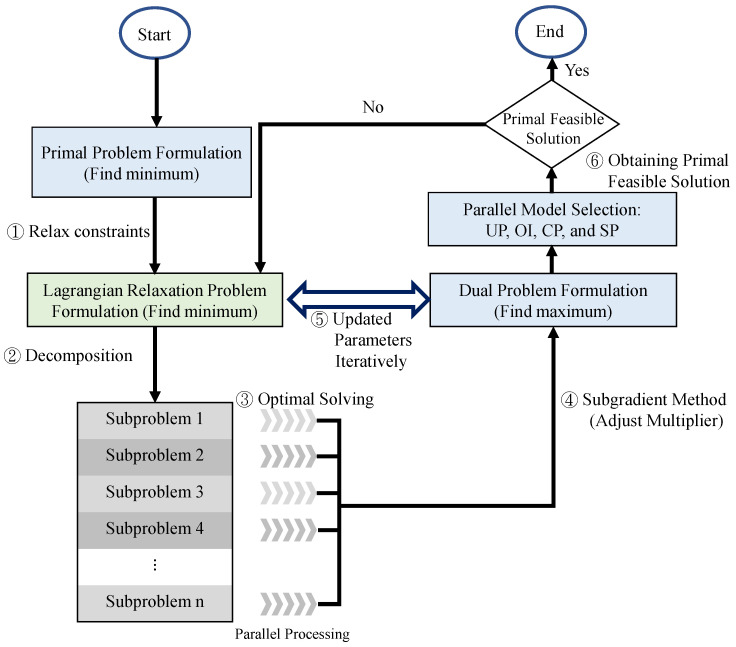
LR-based solution procedures.

The procedure of the LR approach is presented and marked in six steps in Figure 4. If a minimization problem is considered, the solution of the LR approach is the lower bounds. The lower bounds are iteratively improved by adjusting the multipliers set between the LR problem and the dual problem. If there is a feasible solution for the primal problem, then the solution is marked. The gap between the lower bounds and the feasible solutions is calculated for the entire process. The calculations are iteratively repeated until the termination conditions are satisfied. The subgradient optimization method is used for adjusting the multipliers in each iteration to accelerate the convergence of the minimization gap [30].

### 4.1. Procedures of Step 1: Relaxation

Based on the standard form of an optimization problem proposed in Reference [30], the objective function, $Z_{\mathrm{IP}}$, is reformulated as a minimization problem to take from max to min and multiply with a negative sign. The constraints, (C. 3), (C. 6), (C. 7), (C. 9), (C. 10), (C. 12), (C. 14), (C. 15), and (C. 17), have decision variables on both sides of the inequality. They are selected to be relaxed and multiplied by non-negative Lagrangian multipliers. They are added to the objective functions for step 1 in Figure 4, as shown in Equation (2), and denoted as $Z_{\mathrm{LR}}$.

(2) $$\begin{array}{cc} Z_{\mathrm{LR}}=\; & -\sum_{\tau \in T}\sum_{i\in I}\sum_{s\in S}V_{i}a_{\tau is}+\sum_{i\in I}N_{i}b_{i}\\ \; & +\sum_{\tau \in T}\sum_{s\in S}A_{s}x_{\tau s}+\sum_{\tau \in T}\sum_{s\in S}E_{s}y_{\tau s}\\ \; & +\sum_{\pi \in \Pi }\sum_{i\in I}F_{i}\eta_{\pi i}\\ \; & +\sum_{\tau \in T}\sum_{i\in I}\sum_{s\in S}\mu_{\tau is}^{1}\left(a_{\tau is}-x_{\tau s}\right)\\ \; & +\sum_{\tau \in T}\sum_{s\in S}\mu_{\tau s}^{2}\left(\sum_{i\in I}a_{\tau is}D_{i}-x_{\tau s}P_{s}C_{s}\right)\\ \; & +\sum_{\tau \in T}\sum_{s\in S}\mu_{\tau s}^{3}\left(\sum_{i\in I}a_{\tau is}R_{i}-x_{\tau s}M_{s}\right)\\ \; & +\sum_{\tau \in T}\sum_{s\in S}\mu_{\tau s}^{4}\left(x_{\tau s}-x_{(\tau -1)s}-y_{\tau s}\right)\\ \; & +\sum_{i\in I}\mu_{i}^{5}\left(\frac{\gamma_{i}-\sum_{\tau \in T}\sum_{s\in S}a_{\tau is}}{\gamma_{i}}-b_{i}\right)\\ \; & +\sum_{i\in I}\mu_{i}^{6}\left(b_{i}-\gamma_{i}+\sum_{\tau \in T}\sum_{s\in S}a_{\tau is}\right)\\ \; & +\sum_{\tau \in T}\sum_{\pi \in \Pi }\sum_{i\in I}\sum_{s\in S}\mu_{\tau \pi is}^{7}\left(a_{\tau is}-\alpha_{\pi is}\right)\\ \; & +\sum_{\pi \in \Pi }\sum_{i\in I}\sum_{s\in S}\mu_{\pi is}^{8}\left[\left(\alpha_{\pi is}-\alpha_{(\pi -1)is}\right)-\eta_{\pi i}\right]\\ \; & +\sum_{s\in S}\mu_{s}^{9}\left(\sum_{\tau \in T}y_{\tau s}-\frac{T}{2}\right). \end{array}$$

Afterwards, the optimization problem can be reformulated as

$$\begin{array}{cc} \mathrm{Objective}\;\mathrm{function}\text{:}\\ \min\; & \;Z_{\mathrm{LR}}\\ \mathrm{subject}\;\mathrm{to}\text{:}\; & \;(C.\;1),(C.\;2),(C.\;4),(C.\;5),(C.\;8),(C.\;11),(C.\;13),(C.\;16), \end{array}$$

where $a_{\tau is}=0$ or 1, $x_{\tau s}=0$ or 1, $y_{\tau s}=0$ or 1, $b_{i}=0$ or 1, $\alpha_{\pi is}=0$ or 1, and $\eta_{\pi i}=0$ or 1.

A fewer number of remaining constraints are separated and solved in the sub-problems related to the decision variables, accordingly. By considering a subset of decision variables one at a time, six independent sub-problems are decomposed using the LR problem for Step 2 in Figure 4. The objective function and the remaining constraints are not transformed into a complex problem. The optimal solutions can be determined one by one with Step 3 in Figure 4, parallelly.

### 4.2. Procedures of Step 2 and 3: Decomposition and Solving Sub-Problems

#### 4.2.1. Sub-Problem 1 (Related to $a_{\tau is}$)

By considering the variables related to $a_{\tau is}$, the optimization problem is shown as

(3) $$\begin{array}{cc} \; & \mathrm{Objective}\;\mathrm{function}:\\ \; & \min\;\;\;\;\sum_{\tau \in T}\sum_{i\in I}\sum_{s\in S}\left(\begin{array}{cc} \; & -V_{i}+\mu_{\tau is}^{1}+\mu_{\tau s}^{2}D_{i}+\mu_{\tau s}^{3}R_{i}\\ \; & -\frac{\mu_{i}^{5}}{\gamma_{i}}+\mu_{i}^{6}+\left(\sum_{\pi \in \Pi }\mu_{\tau \pi is}^{7}\right) \end{array}\right)a_{\tau is}\\ \; & \mathrm{Subject}\;\mathrm{to}:\;\left(C.\;1\right),\left(C.\;2\right),\left(C.\;4\right),\left(C.\;5\right),\;\mathrm{and}a_{\tau is}=0\;\mathrm{or}\;1. \end{array}$$

(3) is a cost minimization problem. The first observation is that the coefficients of (3) can be divided into |T||I||S| sub-problems. If the coefficient of each sub-problem is negative, then the decision variable $a_{\tau is}$ is set to one at index time τ, task *i*, and server *s*. Note that any value for $a_{\tau is}$ is all feasible for constraints (C. 1), (C. 2), (C. 4), and (C. 5).The result is that the minimum objective value is determined. The summation of all the objective values of |T||I||S| sub-problems with the assigned parameter $a_{\tau is}$ is set to one, and the optimal solution is determined. The running time is O(|T||I||S|log|T||I||S|), and the pseudo-code is illustrated as shown in Algorithm 1.

#### 4.2.2. Sub-Problem 2 (Related to $x_{\tau s}$)

By considering the variables related to $x_{\tau s}$, the optimization problem is shown as

(4) $$\begin{array}{cc} \; & \mathrm{Objective}\;\mathrm{function}:\\ \min & \;\;\sum_{\tau \in T}\sum_{s\in S}\left[\begin{array}{cc} \; & A_{s}-\sum_{i\in I}\mu_{\tau is}^{1}-\mu_{\tau s}^{2}P_{s}C_{s}\\ \; & -\mu_{\tau s}^{3}M_{s}+\mu_{\tau s}^{4}-\mu_{(\tau +1)s}^{4} \end{array}\right]x_{\tau s}\;\forall \tau <\left|T\right|\\ \min & \;\;\sum_{\tau \in T}\sum_{s\in S}\left[\begin{array}{cc} \; & A_{s}-\sum_{i\in I}\mu_{\tau is}^{1}-\mu_{\tau s}^{2}P_{s}C_{s}\\ \; & -\mu_{\tau s}^{3}M_{s}+\mu_{\tau s}^{4} \end{array}\right]x_{\tau s}\;\forall \tau =\left|T\right|\\ \mathrm{subject}\;\mathrm{to}: & \;\left(C.\;11\right)\;\mathrm{and}\;x_{\tau s}=0\;\mathrm{or}\;1. \end{array}$$

(4) can be divided into |T||S| sub-problems, as well. If the coefficient corresponding to the sub-problem is negative, the decision variable $x_{\tau s}$ is set to one. For the feasible region (C. 11) of (4), *G* is denoted by at least *G* servers should be switched on at every time τ, which is a policy of the system design. If no coefficient of (4) is less than zero, the minimal objective value of (4) is determined by setting *G* numbers of $x_{\tau s}$ equal to one. On the contrary, the numbers are set to zero. The running time is O(|T||S|), and the pseudo-code is demonstrated as shown in Algorithm 2.
**Algorithm 1** Sub-problem 1
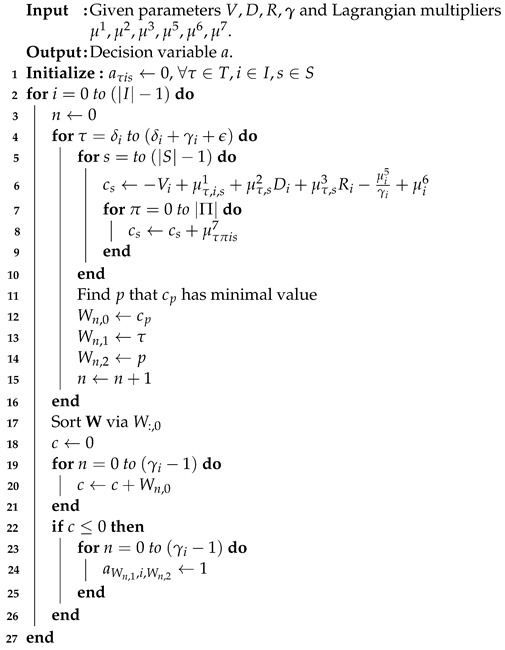


#### 4.2.3. Sub-Problem 3 (Related to $y_{\tau s}$)

By considering the variables related to $y_{\tau s}$, the optimization problem is shown as

(5) $$\begin{array}{cc} \mathrm{Objective}\;\mathrm{function}: & \\ \min\;\; & \;\sum_{\tau \in T}\sum_{s\in S}\left(E_{s}-\mu_{\tau s}^{4}+\mu_{s}^{9}\right)y_{\tau s}\\ \mathrm{subject}\;\mathrm{to}: & \;\left(C.\;13\right)\;\mathrm{and}\;y_{\tau s}=0\;\mathrm{or}\;1. \end{array}$$

(5) can also be divided into |T||S| sub-problems. When the corresponding coefficient is less than zero, the decision variable $y_{\tau s}$ is set to one. Note that any value for $y_{\tau s}$ is all feasible for constraints (C. 13). The running time is O(|T||S|), and the pseudo-code is illustrated as shown in Algorithm 3.
**Algorithm 2** Sub-problem 2
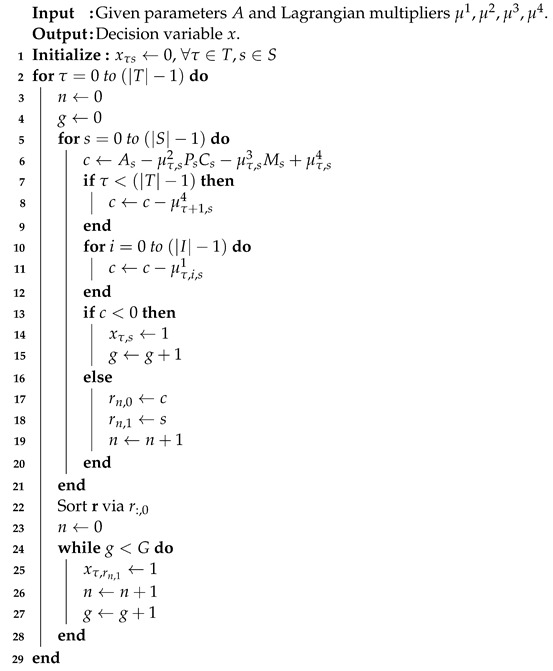


**Algorithm 3** Sub-problem 3

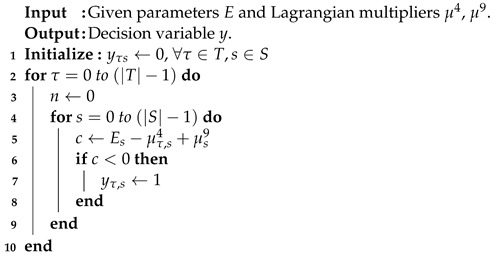



#### 4.2.4. Sub-Problem 4 (Related to $b_{i}$)

By considering the variables related to $b_{i}$, the optimization problem is shown as

(6) $$\begin{array}{cc} \mathrm{Objective}\;\mathrm{function}: & \\ \min\;\; & \;\sum_{i\in I}\left(N_{i}-\mu_{i}^{5}+\mu_{i}^{6}\right)b_{i}\\ \mathrm{subject}\;\mathrm{to}: & \;\left(C.\;8\right)\;\mathrm{and}\;b_{i}=0\;\mathrm{or}\;1. \end{array}$$

(6) can be divided into |I| sub-problems. In each sub-problem, the decision variable $b_{i}$ is set to one, whereas the coefficient is less than zero. Subject to constraint (C. 8), the maximum amount of $b_{i}$ set to one should not be greater than K|I|. The pseudo-code is demonstrated as shown in Algorithm 4 with the running time O(|I|log|I|).
**Algorithm 4** Sub-problem 4
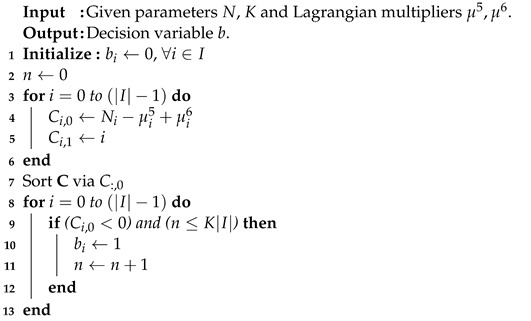


#### 4.2.5. Sub-Problem 5 (Related to $\alpha_{\pi is}$)

By considering the variables related to $\alpha_{\pi is}$, the optimization problem is shown as

(7) $$\begin{array}{cc} \mathrm{Objective}\;\mathrm{function}: & \\ \min & \;\sum_{\pi \in \Pi }\sum_{i\in I}\sum_{s\in S}\left(-\sum_{\tau \in T}\mu_{\tau \pi is}^{7}+\mu_{\pi is}^{8}-\mu_{(\pi +1)is}^{8}\right)\alpha_{\pi is},\;\forall \pi <\left|\Pi \right|\\ \min & \;\sum_{\pi \in \Pi }\sum_{i\in I}\sum_{s\in S}\left(-\sum_{\tau \in T}\mu_{\tau \pi is}^{7}+\mu_{\pi is}^{8}\right)\alpha_{\pi is},\;\forall \pi =\left|\Pi \right|\\ \mathrm{subject}\;\mathrm{to}: & \;\left(C.\;16\right)\;\mathrm{and}\;\alpha_{\pi is}=0\;\mathrm{or}\;1. \end{array}$$

The solution process of (7) is similar to that of (4). (7) is divided into |Π||I||S| sub-problems. The decision variable $\alpha_{\pi is}$ is set to one if the coefficients corresponding to index migration time π, task *i*, and server *s* of the sub-problem are negative. The difference between (4) and (7) is the feasible region, constraint (C. 16); that is, $\alpha_{\pi is}$ is set to one at the server *s* which has negative coefficients for specific index migration time π and task *i*. The running time is O(|Π||I||S|), and the pseudo-code is illustrated as shown in Algorithm 5.
**Algorithm 5** Sub-problem 5
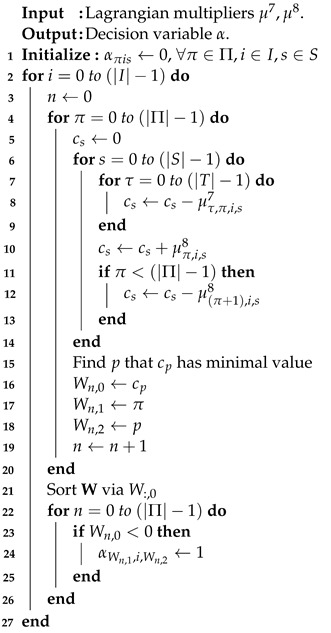


#### 4.2.6. Sub-Problem 6 (Related to $\eta_{\pi i}$)

By considering the variables related to $\eta_{\pi i}$, the optimization problem is shown as

(8) $$\begin{array}{cc} \mathrm{Objective}\;\mathrm{function}: & \\ \min & \;\sum_{\pi \in \Pi }\sum_{i\in I}\left(F_{i}-\sum_{s\in S}\mu_{\pi is}^{8}\right)\eta_{\pi i}\\ \mathrm{subject}\;\mathrm{to}: & \;\eta_{\pi i}=0\;\mathrm{or}\;1. \end{array}$$

The objective function of (8) is also a cost minimization problem that can be divided into |Π||I| sub-problems. If the coefficient of each sub-problem is negative, then the decision variable $\eta_{\pi i}$ is set to one at index migration interval π and task *i*. The minimum objective value is determined by the summation of all the objective values of |Π||I| sub-problems with the assigned parameter $\eta_{\pi i}$ set to one. The running time is O(|Π||I|), and the pseudo-code is illustrated as shown in Algorithm 6.
**Algorithm 6** Sub-problem 6
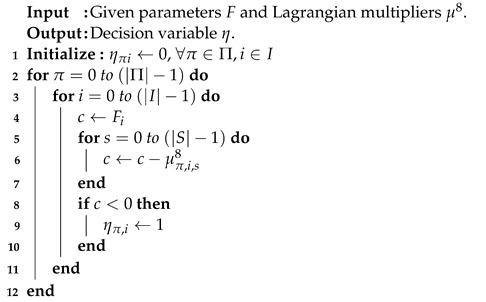


### 4.3. Procedure of Step 4 and 5: Dual Problem and the Subgradient Method

**Figure 5:**
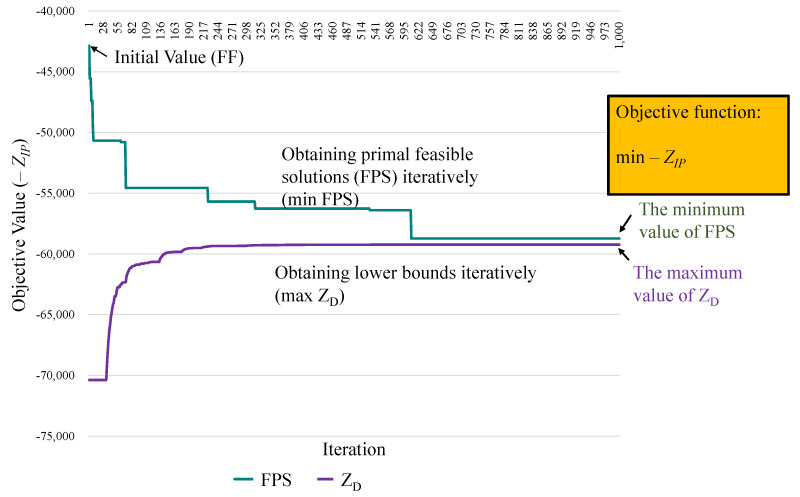
Obtaining primal feasible solutions and the lower bound (LB) [20].

Based on the weak Lagrangian duality theorem [29], the multiples are all non-negative values; that is, $\mu_{\tau is}^{1},\mu_{\tau s}^{2},\mu_{\tau s}^{3},\mu_{\tau s}^{4},\mu_{i}^{5},\mu_{i}^{6},\mu_{\tau \pi is}^{7},\mu_{\pi is}^{8},\mu_{i}^{9}\geq 0,\forall \tau \in T,\forall \pi \in \Pi ,\forall i\in I,$ and ∀s∈S. The objective value of the LR problem, $Z_{\mathrm{LR}}$, is the lower bound of the primal problem, $Z_{\mathrm{IP}}$. Construct the following dual problem $Z_{D}$ based on the LR problem to calculate the tightest boundary of steps 4 and 5 in Figure 5, where the dual problem (9) is shown as [31]

(9) $$\begin{array}{cc} \mathrm{Objective}\;\mathrm{function}: & \\ \max & \;Z_{D}\\ \mathrm{subject}\;\mathrm{to}: & \;\mu^{1},\mu^{2},\mu^{3},\mu^{4},\mu^{5},\mu^{6},\mu^{7},\mu^{8},\mu^{9}\geq 0. \end{array}$$

There are several methods to solve the dual problem (9). The most popular one of them is the subgradient method proposed in Reference [28,29]. First, define the multiplier vector of *k*-th iteration, $\pi^{k}=(\mu^{1,k},\mu^{2,k},\mu^{3,k},\mu^{4,k},\mu^{5,k},\mu^{6,k},\mu^{7,k},\mu^{8,k},\mu^{9,k})$, and let it to be iteratively updated by $\pi^{k+1}=\pi^{k}+t^{k}{\;s}^{k}$, where *k* is the number of iterations. The vector $s^{k}$ is the subgradient of $Z_{D}$ of the *k*-th iteration which represents the directions to the optimal solutions. The step size $t^{k}$ is determined by $t^{k}=\lambda \frac{\left(Z_{\mathrm{IP}}^{*}-Z_{D}\left(\pi^{k}\right)\right)}{\left|\right|s^{k}{\left|\right|}^{2}}$, where $Z_{\mathrm{IP}}^{*}$ is an objective value of the primal problem, and λ is a constant, 0≤λ≤2. The optimal solutions of the LR and dual problems for each iteration are determined by solving the sub-problems and iteratively updating the multipliers by using the subgradient method, respectively.

### 4.4. Procedure of Step 6: Obtaining the Primal Feasible Solutions

The LR and Subgradient methods determine the theoretical bounds and provide some hints toward the primal feasible solutions. Based on the sensitivity properties in Reference [32], the Lagrangian multipliers are represented by weights, which are also known as the cost per unit constraint violation for the objective value improvement rate. In observing the primal feasible region of the primal problem, the solutions must satisfy all the constraints. A set of primal feasible solutions to $Z_{\mathrm{IP}}$ is a subset of the solutions to $Z_{D}$. For Step 6 in Figure 4, the following four model selections based on the properties of Lagrangian multipliers are proposed for obtaining primal feasible solutions, parallelly.

#### 4.4.1. Urgent and Penalty (*UP*) Involved

Task assignment and scheduling are considered in combination in the first step for obtaining primal feasible solutions. A heuristic is proposed to determine the order of tasks sorted by the level of significance. An index in Equation (10), urgent and penalty (*UP*), is created by analyzing the coefficients of SUB4 with the penalty value. A brief analysis of the coefficient of SUB4 implies the significance of tasks. However, the multipliers, $\mu_{i}^{5}$ and $\mu_{i}^{6}$, are represented as the requested processing time’s satisfaction variables and assigned time slot. $N_{i}$ is the penalty of a task that occurs if the requested processing time is not satisfied. The orchestrator is allowed to determine the crucial decisions of rejection and each task’s assigned processing time.

According to constraints (C. 6) and (C. 7), the decision variable $b_{i}$ is set to zero if the processing time required by task *i* is satisfied. Otherwise, it is set to one if the requirement is not fulfilled. Thus, combine corresponding multipliers $\mu_{i}^{5}$ and $\mu_{i}^{6}$ with the penalty of rejection $N_{i}$ as an index of processing priority.

(10) $$UP_{i}=N_{i}-\mu_{i}^{5}+\mu_{i}^{6}\;\forall i\in I.$$

#### 4.4.2. Optimization-Based Significance Index (*OI*)

Based on the previous index, the significance index (*OI*) is also derived from the two multipliers $\mu_{i}^{5}$ and $\mu_{i}^{6}$, as shown in (11). The stages for considering $\mu_{i}^{5}$ and $\mu_{i}^{6}$ can be observed in constraints (C. 6) and (C. 7). These two constraints are satisfied under the conditions in which a task requests appropriate assignments. The decision variables $b_{i}$ in constraints (C. 6) and (C. 7) are both set to zero, which indicates that all the requested processing time slots are assigned. For each iteration, the absolute value of the subtraction of $\mu_{i}^{5}$ and $\mu_{i}^{6}$ is used to evaluate the task significance of assignments properly.

(11) $$OI_{i}=\left|\mu_{i}^{6}-\mu_{i}^{5}\right|\;\forall i\in I.$$

#### 4.4.3. Cost Performance (*CP*)

This heuristic approach is proposed to establish the concept of the cost performance (*CP*) ratio of the tasks. The *CP* ratio is defined as the ratio of the task values per requested demand, as shown in (12). This heuristic concept is derived from identifying the tasks with the maximum CP value that contributes to the most objective values. The *CP* values are used to determine the tasks that have to be performed or dropped. The tasks with higher *CP* values than those with lower *CP* values are selected to contribute to the objective values. The tasks that reflect the high *CP* values in objective functions are accurately identified in the method.

(12) $$CP_{i}=\frac{V_{i}}{D_{i}R_{i}}\;\forall i\in I.$$

#### 4.4.4. Shortest-Path-Based Significance Index (SP)

**Figure 6:**
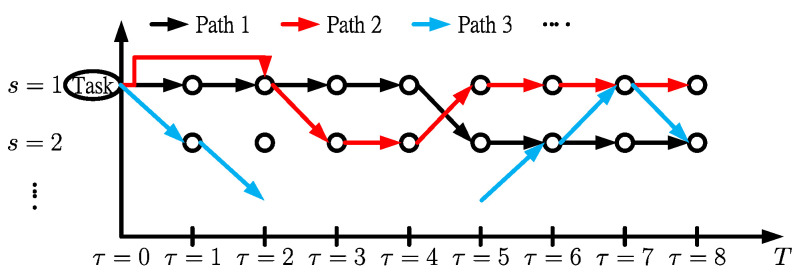
Tree structure of the *SP*.

We can find three independent dimension problems with time slot τ, task *i*, and server *s* from the observation of objective function in the primal problem. The aggregation of the assigned tasks does not exceed the capacity of the servers. Furthermore, a tree data structure can be represented as a set of linked nodes for task *i* in which the root is the starting time of the task assignment. The branches are the assigned servers after a global analysis of the objective function. The destination is the deadline related to the servers. Suppose there is any path available from the root to the destination. In that case, the task assignment series can be determined, as shown in Figure 6.

**Figure 7:**
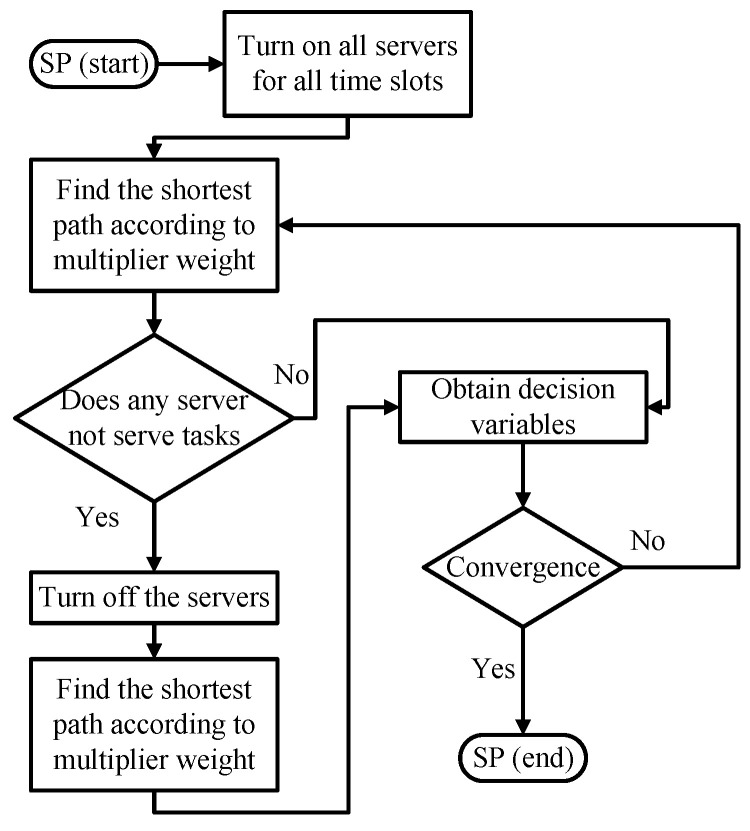
Flowchart of the *SP*.

The Border Gateway Multicast Protocol (BGMP) and a scalable multicast routing protocol use heuristics to select the global root of the delivery tree [33]. We develop source-specific shortest-path distribution branches to supplant the shared tree. The arc weights are the coefficients of SUB1. The arc weights of migration are also considered. Moreover, the problem is transformed into a path length-restricted shortest path problem. We identify the paths with weights from the task arrival to departure. Furthermore, the shortest path problem can be effectively solved using the Bellman-Ford algorithm [34]. Link constraints with server CPU and memory capacities are the restrictions set on using links to form a routing tree [34]. The path constraints are the restrictions to initialize the time slot and select the branches related to the servers switched on or off. The detailed flowchart is shown in Figure 7, where the decision variables are fine-tuned to feasible from the dual problem to the *SP* model.

## 5. Computational Experiments and Numerical Results

### 5.1. Methodology and Experimental Environments

Some experiment scenarios are designed for an orchestrator for network operation to evaluate the performance based on communication and computational perspectives. We then explain how the experimental cases lead to a real-life situation and discuss how the results changed due to various using cases. The experimental environment is initialized for resource access control, scheduling, and migration models embedded into an orchestrator in C-RAN. The algorithms are constructed and implemented to analyze the heuristics’ quality for conducting performance evaluations in several simulation cases. Our experiments are developed in C++ and implemented in a personal computer with a quad-core processor, 8 GB RAM, and Ubuntu operating system version 14.04. The experimental parameters are listed in Table 4. The given amount of task traffic loads and arrival time slots are randomly generated and fixed for each evaluation case. The solution to the dual problem is denoted as $Z_{D}$. The $Z_{D}$ values are the lower bounds of the primal solutions. The dual solutions are not feasible for the primal problem because some constraints are relaxed. Therefore, the primal objective value is not always lower than the $Z_{D}$ value, referred to as the lower bound. However, a primary research goal is to obtain a feasible solution. For example, Figure 5 presents an experimental case. The green curve represents the process of obtaining the primal feasible solutions iteratively. The objective is to identify the minimum value of the primal problem (min PFS). Then, the values of $Z_{D}$ are determined using the subgradient method to iteratively obtain the tightest LB (max $Z_{D}$), represented by the purple line. The first step is to identify the initial solutions determined by the *FF* algorithm [35]. The second step is to determine the primal feasible solutions of the proposed strategies by *UP*, *OI*, *CP*, and *SP*. The performance metric, the gap, is used to evaluate the solution quality which is defined as $\mathrm{gap}=\frac{\left|PFS-Z_{D}\right|}{\left|Z_{D}\right|}\times 100\%$, where *PFS* is the primal feasible solution of proposed strategy.

Subsequently, several scenarios are designed for performance evaluation based on different communication and computational perspectives. The experimental results are used to analyze the objective values that influence decisions in strategies.

### 5.2. Numerical Results

#### 5.2.1. Uniform Traffic Load

**Figure 8:**
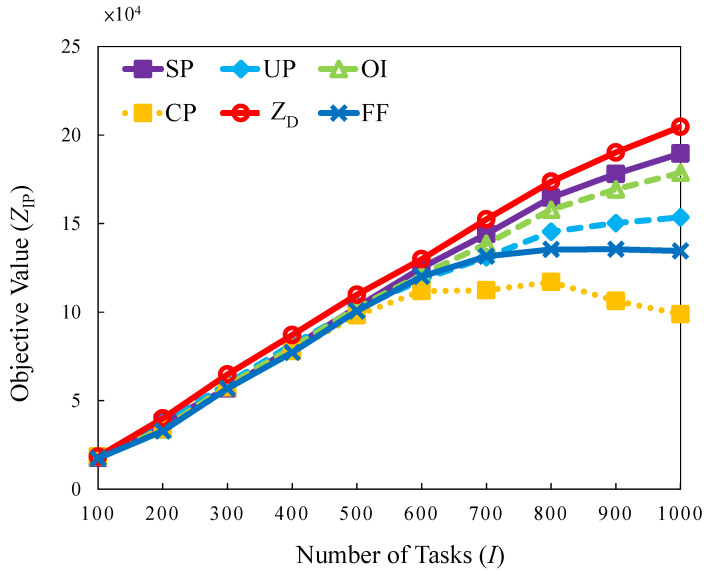
Objective value versus different number of tasks in uniform traffic load.

The experiment is designed to consider task arrivals with a uniform distribution, which is interpreted as normal traffic for daily cases in a regular manner, as shown in Table 4. First, this case examines the objective values trend with the number of tasks as the control variable. Figure 8 displays the result; the objective value increases along with the number of tasks. Compared with the curves of *FF*, *SP*, *OI*, and *UP* to demonstrate how high the performance than of the proposed *FF* algorithm when the number of tasks is higher than 700. The reason is that the dropping penalty is considered due to the number of tasks that is beyond the system capacity.

Method *CP* exhibits the worst performance. The decreasing trends in this method are lower than those mentioned above or the *FF* method for tasks. In brief, better resource allocation and scheduling strategies are jointly considered for determining the level of task significance through a near-optimization-based solution with *SP*, *OI*, and *UP* for maximizing objective values. The *SP* is the mostly near-optimal strategy with the highest number of feasible solutions, and the minimum solution gap (3.74%∼12.19%).

#### 5.2.2. Bursty Traffic Load

**Figure 9:**
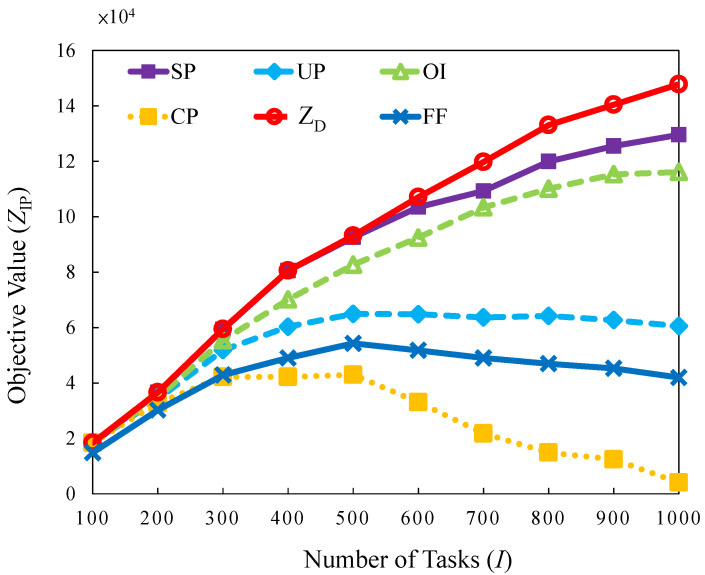
Objective value versus different number of tasks in bursty traffic load.

A burst pattern in which a substantial number of tasks arrived in a short period is emulated. This scenario examines how the burst task arrivals influence the objective values. The results are shown in Figure 9, revealing that the difference between the strategies is considerable. *SP*, *OI*, and *UP* perform better than *FF* because they have a sufficient buffer time to reassign tasks or conduct migrations to maximize the values of tasks selected to serve within a limited server capacity. *FF* performs worse than other methods because it has no mechanism to switch servers off and no buffer time to reassign tasks. The results obtained by observing Figure 9 reveal that *CP* exhibits the lowest performance. In this case, the tasks are not assigned to appropriate servers. It causes a higher number of blocked tasks and correspondingly generates penalties that reduce the total revenue and lower the objective value. *SP* provides the most optimal solutions compared with other strategies. The gap is 0% for 100 to 500 tasks, thus indicating that optimal solutions are determined. In other cases, the values of the gap are 3.33% to 12.19%.

#### 5.2.3. Evaluation of Allowed Waiting (Buffer) Time

**Figure 10:**
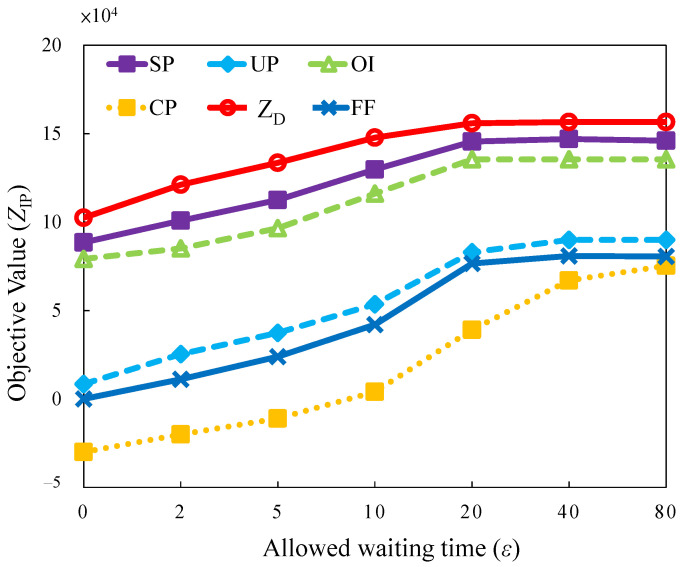
Objective value versus different allowed waiting time.

The allowed waiting time for each task is referred to as delay tolerance. The delay tolerance length is related to the type of applications requested by tasks, such as web browsing, file transfer, or video streaming. The correlation between the delay and objective value is considered in this case. In general, the short tolerance delay for a task implies less flexibility for task assignment and scheduling. It results in a higher blocking probability than in the case with a longer tolerance delay. However, the results obtained by analyzing in Figure 10 reveal that different waiting times and bursty traffic loads are designed in combination with tolerance length. *SP*, *OI*, and *UP* exhibit increasing trends in the saturation region in the allowed buffer time from 20 to 80.

The reasons for the increasing trend are: (i) The tasks are assigned to servers at a long time slot; (ii) the burst traffics arrive after a long buffer waiting time for aggregation within the long time available for task migration. The longer the waiting time, the higher is the revenue incurred. The delay tolerance creates long time-space by manipulating or scheduling tasks. In summary, an operator should set an appropriate QoS metric for SLA embedded into an orchestrator to recommend user applications with the maximum acceptable delay tolerance.

#### 5.2.4. Evaluation of Server Cost Rate

This experiment evaluates the effect of the increasing server cost rate. Various servers with different levels of capacities are deployed in a data center. It is impossible to replace all the servers when the data center upgrades [19]. Different levels of servers have diverse costs and capacities. Maintaining all the servers homogeneously in a data center all the time is difficult. The results of a cluster trace published by Google indicate that the servers are heterogeneous [36].

Based on the situation, the servers with various capacities and costs were emulated. The base server generates a growing cost rate with the minimum capacity and corresponding cost. For example, the value of 1.2 implies that the cost and capacity are 1.2 times higher than the base server. The diversities of levels for servers are emulated in the experimental environments. They are lowest levels cost rate of servers 0.1 up to the highest levels cost rate of servers 1.3.

**Figure 11:**
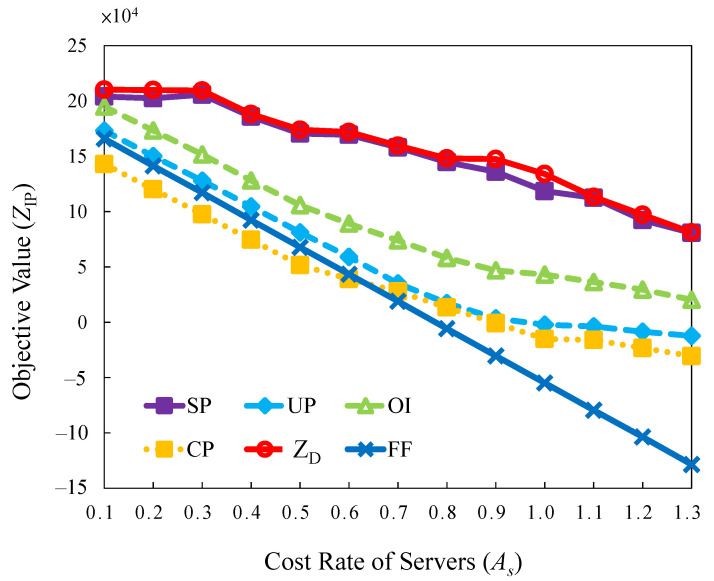
Objective value versus different cost rate.

The curves in Figure 11 exhibit trends similar to those of the previous cases’ task penalty section. Increasing the cost rate causes a decrease in the objective value. However, the cost and objective values are not represented linearly in the regular descending order in the *SP*, *OI*, *UP*, and *CP* strategies.

The results are that changing the cost rates in different servers provides alternative decisions to switch on the cost-efficient servers. For high server cost rates, the curves of *SP*, *OI*, *UP*, and *CP* are moderate compared with that of *FF*. Concerning active servers in the serving status, the *SP*, *OI*, *UP*, and *CP* strategies have cost-effectiveness and achieve higher objective values than *FF*. The results also reveal that *SP* is the best strategy with the highest feasible solutions and the minimum solution gap for most server cost configurations.

### 5.3. Time Complexity Analysis

The time complexities of the methods, such as *UP*, *OI*, *CP*, and *SP*, are analyzed for comparison to the initial method, *FF*. The corresponding data is followed. The time complexity of the proposed LR-based solutions (*UP*, *OI*, *CP*, and *SP*) is O(N|T||I||S|log|T||I||S|). The Lagrange dual solution is determined by Sub-problems (3)–(8). The time complexity is the worst of sub-problems, which is O(|T||I||S|log|T||I||S|) of SUB1 in parallel processing by divide-and-conquer algorithms. Each sub-problem required O(|T||I||S|log|T||I||S|) time minimum coefficient among servers. The Lagrange dual solutions could be obtained after a maximum number of iterations *N*, and the time complexity is O(N|T||I||S|log|T||I||S|). The convergence is achieved in a small number of iterations (about 600) shown in Figure 5. However, the time complexity of the Brute force is $O(2^{{\left|T\right|}^{3}{\left|I\right|}^{3}{\left|S\right|}^{3}\left|\Pi \right|})$ with the total combination of decision variables. The complexity of *FF* is O(|T||I||Π||S|) with the sequential screening method. Furthermore, the proposed algorithms (*UP*, *OI*, *CP*, and *SP*) can be set as the output results at any complete iteration. Thus, the time can be controlled in practice.

## 6. Conclusions

In this paper, the research scope is proposed for an Internet service provider to optimize system resource utilization for network operation. The proposed system architecture with a resource orchestrator is a shared and connected resource processing VM pool inspired by a cloud computing environment. The orchestrator is a coordinator through access control to make resource scheduling and migration decisions in network operational stages. A mathematical model is formulated to solve network operational problems with elastic access control, scheduling, and migration mechanisms. The motivation and objective are extended from the previous work [20] dealing with a network operation problem, not only from a resource planning perspective. The operation is divided into slices with many time slots in the evaluation period—additionally, the available hosts scheduling with migration to adjust into active hosts or servers. The operation costs are reflected in the objective function. In this situation, the extra migration time is calculated as a trade-off problem to migrate or not. The objective function includes assigned host on-off and migration costs with the proposed scheduling heuristic among allocated time slots. Designing management strategies is created by satisfying users’ QoS and increasing resource pool utilization. Some cases emulated as realistic environment datasets, such as uniform or bursty traffic loads, in the experiments. It is designed for performance evaluation of an orchestrator for network operation based on different communication and computational perspectives. Accordingly, the realistic dataset may be helpful in fine-tuning to analyze values in the system operation stages or specific cases. The proposed heuristics can then iteratively obtain the primal feasible solutions to minimize the solutions’ quality effectively. The performance of the proposed heuristics (*SP*, *UP*, and *OI*) is compared with the baseline, classical methods (*FF* and *CP*). Regarding our numerical results, the *SP* method outperforms the other methods in increasing uniform/bursty traffic load, decreasing allowed waiting time, and rising cost rate. It can achieve near-optimal system revenue by obtaining decisions in sliced networks. The composition of decisions with task access control, resource allocation, scheduling, and migrations are significant in efficiently and effectively network operation.

## Figures and Tables

**Table 1:** Proposed model comparisons with literature.

	Access Control	Resource Scheduling	Migration	Computing Perspective	Communication Perspective
[3,6,8,13,16]		✔	✔	✔	✔
[4,6,13,16]		✔	✔	✔	
[6,8,13,16,19]		✔			✔
[11,12,15,21]			✔	✔	✔
[11,12,13,14,24]	✔			✔	✔
[9]		✔			✔
[20]	✔	✔		✔	✔
Proposed model	✔	✔	✔	✔	✔

**Table 2:** Given parameters.

Notation	Description
*T*	A set of time slots, T={1,2,…,τ,…,|T|}
*I*	A set of VMs with VNFs or called tasks, I={1,2,…,i,…,|I|}
*S*	A set of physical servers in C-RAN, S={1,2,…,s,…,|S|}
Π	A set of time slots in large time scale, Π={1,2,…,π,…,|Π|}
$t_{\tau }$	System time of the time index τ, $t_{\left|T\right|}=t_{\left|\Pi \right|}$
$V_{i}$	Reward rate of task *i*, which is a function of CPU or memory requirements, ∀i∈I
$N_{i}$	Penalty of task *i* if it is rejected when the requirement is not satisfied, ∀i∈I
$A_{s}$	Setup cost rate of the *s*-th server, ∀s∈S
$E_{s}$	Reopen cost of the *s*-th server, ∀s∈S
$F_{i}$	Migration cost of task *i*, ∀i∈I
$\delta_{i}$	The system time that task *i* is arrived, ∀i∈I
$\gamma_{i}$	Processing time required by task *i*, which means processing time of long-term statistics (length of time per task), ∀i∈I
ϵ	Allowed waiting time for a task when it arrives and is complete
*K*	Total task-blocking rate
$D_{i}$	Total amount of CPU processing speed (GHz) required by task *i*, ∀i∈I
$P_{s}$	Number of CPU cores installed in server *s*, ∀s∈S
$C_{s}$	Processing capability (GHz) of each CPU core in server *s*, ∀s∈S
$R_{i}$	Total amount of RAM required by task *i*, ∀i∈I
$M_{s}$	RAM capacity of server *s*, ∀s∈S
*G*	The minimum number of servers that is switched on

**Table 3:** Decision variables.

Notation	Description
$a_{\tau is}$	Binary variable, 1 if task *i* is assigned to server *s* in time slot τ and 0 otherwise, ∀τ∈T, ∀i∈I, ∀s∈S
$x_{\tau s}$	Binary variable, 1 if server *s* is switched on in time slot τ and 0 otherwise, ∀τ∈T, ∀s∈S
$y_{\tau s}$	Binary variable, 1 if server *s* being powered-on in time slot τ when it was powered-off in previous time τ−1 and 0 otherwise, ∀τ∈T, ∀s∈S
$b_{i}$	Binary variable, 1 if task *i* is rejected and 0 otherwise, ∀i∈I
$\alpha_{\pi is}$	Binary variable, 1 if task *i* is assigned to server *s* in time slot π and 0 otherwise, ∀π∈Π, ∀i∈I, ∀s∈S
$\eta_{\pi i}$	Binary variable, 1 if task *i* is migrated in time slot π and 0 otherwise, ∀π∈Π, ∀i∈I

**Table 4:** Parameters for computational experiments.

Given Parameter	Value
Number of time slots (|T|)	100
Number of tasks (|I|)	400
Number of servers (|S|)	4
Processing capability ($P_{s}C_{s}$) (GHz)	(1+s/8)×250, for s=0,1,2,3
RAM capacity ($M_{s}$) (GBytes)	(1+s/8)×250, for s=0,1,2,3
Setup cost rate ($A_{s}$)	$(P_{s}C_{s}+M_{s})\times 0.2$
Reopen cost ($E_{s}$)	$A_{s}\times 1.5$
Migration cost ($F_{i}$)	100
Reward rate ($V_{i}$)	$V_{i}∼U\left(1,100\right)$
CPU processing capability ($D_{i}$) (GHz)	$D_{i}∼U\left(1,50\right)$
Total required RAM ($R_{i}$) (GBytes)	$R_{i}∼U\left(1,50\right)$
The system time that the task *i* is arrived ($\delta_{i}$) (sec.)	$\delta_{i}∼U\left(1,|T|-1\right)$
Allowed waiting time (ϵ) (sec.)	10

## Data Availability

Not applicable.
